# Development and psychometric validation of a preschool social-emotional competence scale in China

**DOI:** 10.3389/fpsyg.2026.1729966

**Published:** 2026-04-08

**Authors:** Lingjuan Zeng, Tianyu Tong, Danting Qin, Shanshan Meng, Xiaomin Zhu, Caiyun Meng, Chaozheng Wei

**Affiliations:** 1 College of Education Science, Nanning Normal University, Nanning, Guangxi, China; 2 The People’s Liberation Army Joint Logistics Support Force No. 923 Hospital Kindergarten, Nanning, Guangxi, China

**Keywords:** preschool, reliability, scale development, social-emotional competence, validity

## Abstract

**Introduction:**

Social-emotional competence is a core developmental capacity in early childhood, yet culturally grounded assessment tools for Chinese preschoolers remain limited. This study aimed to develop and validate the Preschool Social-Emotional Competence Scale for children in Guangxi Zhuang Autonomous Region, China.

**Methods:**

Guided by social-emotional competence theory and a culturally responsive framework, the scale was developed through literature review, qualitative interviews, expert consultation, and pilot testing. Psychometric properties were examined using caregiver reports for 1,666 preschoolers aged 3–6 years (889 boys, 53.36%; 777 girls, 46.64%).

**Results:**

Exploratory factor analysis supported a 21-item, four-factor structure comprising Emotion and Self-recognition, Daily Habits, Social Interaction, and Bullying Counteraction, explaining 71.67% of the total variance. Confirmatory factor analysis showed good model fit (χ^2^/df = 3.86, GFI = 0.92, CFI = 0.95, IFI = 0.95, TLI = 0.94, RMSEA = 0.06). The scale demonstrated satisfactory convergent and discriminant validity, strong internal consistency (Cronbach’s α = 0.94; subscales α = 0.76–0.94; McDonald’s ω = 0.94; subscale ω = 0.86–0.94), and measurement invariance across gender. Girls scored higher than boys on Emotion and Self-recognition and Daily Habits, whereas no significant differences were found by only-child status. Children from families with lower subjective socioeconomic status scored lower across all domains of social-emotional competence.

**Discussion:**

The Preschool Social-Emotional Competence Scale is a valid and developmentally appropriate instrument for assessing social-emotional competence in typically developing Chinese preschoolers aged 3–6 years. It provides a practical tool for educators and researchers and may help inform targeted early interventions.

## 1 Introduction

Social-emotional competence (SEC) is an integrated capability to manage emotions in pursuit of personal or collective goals, to form and maintain supportive interpersonal relationships, and to make responsible decisions when addressing social problems (CASEL, 2020). Compared with the relative stability of cognitive ability, SEC is environment-sensitive and malleable through education and cultivation (Liu and Liang, 2021); it originates in infancy and develops rapidly during the preschool years (Wang et al., 2023).

Research on preschoolers’ social-emotional competence (P-SEC) in China has largely consisted of translating, revising, and norming foreign instruments, with relatively little theory development or construct validation grounded in local contexts. Because preschoolers’ limited cognitive capacity precludes valid independent self-report, assessments typically rely on teacher or primary caregiver reports or on preschool/kindergarten records. Commonly used tools include the Strengths and Difficulties Questionnaire (SDQ) for children ages 3–6, Gresham and Elliott’s Social Skills Rating System (SSRS), and emotion-matching tasks developed by Morgan et al. Although these early-development measures cover aspects of social competence and emotional maturity, they are used primarily as comprehensive appraisals of children’s overall development rather than as dedicated measures of social-emotional competence (Hao et al., 2024).

Building on a Yale-developed assessment of early childhood social and emotional development, Chinese scholars translated and localized the instrument to create the Chinese Inventory of Social and Emotional Development for preschoolers and established standardized norms. The instrument comprises four domains—Externalizing, Internalizing, Dysregulation, and Competence—spanning 19 factors and 146 items, and it demonstrates good psychometric properties (reliability and validity); it can serve as a common tool for profiling social and emotional development among urban preschoolers in China (Wang et al., 2009). Nevertheless, the measure emphasizes problem behaviors and contains a large number of items, which increases the risk of respondent fatigue and limits its practicality. Given the pronounced subjectivity and cross-cultural variability of social-emotional competence, assessment outcomes are influenced by children’s cognitive developmental level, collectivist cultural orientations, and prevailing social norms.

Early childhood education philosophies and implementation priorities differ across contexts. In the United States, Developmentally Appropriate Practice (DAP) promoted by National Association for the Education of Young Children (2020), together with several social-emotional learning frameworks, emphasizes play-based pedagogy, child agency, and self-expression. Chinese policy documents (Ministry of Education of the People's Republic of China, 2012) stress order and collectivism while likewise identifying play as the fundamental mode of activity. Given the pronounced cultural relevance and contextual sensitivity of social-emotional competence—and its dependence on social norms and children’s cognitive development—assessment frameworks and scales should be developed within the local cultural milieu (Qu and Chen, 2023) and attuned to stage- and context-specific emphases (Ma, 2024).

Enhancing early SEC is critical for later academic success and positive behavioral outcomes (Martinsone et al., 2022), improving non-cognitive abilities such as self-efficacy, belonging, and social adaptation; high SEC is protective of mental health (Zhang et al., 2023; Zhao et al., 2024). This study aimed to develop a culturally and developmentally appropriate scale for assessing social-emotional competence in typically developing Chinese preschoolers and to rigorously evaluate its psychometric properties.

## 2 Scale development: sources and process

### 2.1 Theoretical foundation

Building on prior empirical work and qualitative interview findings, we developed an initial scale of preschoolers’ social-emotional competence and sought content review from experts with more than 10 years of professional experience in early childhood education or psychology. In the scholarly literature, two major approaches to the construct are evident. First, the Collaborative for Academic, Social, and Emotional Learning (CASEL), grounded in emotional intelligence and shaped by educational practice from preschool through high school, proposes a comprehensive framework (Elias et al., 1997). Subsequent iterations culminate in the five interrelated core competencies of the CASEL Wheel—self-awareness, self-management, social awareness, relationship skills, and responsible decision-making (CASEL, 2013, 2020)—which emphasize the integrated interplay of emotion recognition, relationship building, and ethical decision-making, offering a cognitively and behaviorally coordinated pathway for understanding early competence development. Second, the Organization for Economic Co-operation and Development (OECD) addresses issues of conceptual overlap within CASEL (e.g., social awareness vs. social skills) and cross-cultural adaptability by advancing an assessment framework for adolescents derived from the Big Five model: task performance, emotional regulation, open-mindedness, collaboration, engaging with others (sociability), and resilience (Eva, 2021; Walton et al., 2023). In the International Early Learning and Child Well-Being Study, the OECD further refines preschool social-emotional competence into dimensions such as empathy (emotion recognition and understanding), interpersonal trust (confident help-seeking), and prosocial, non-disruptive behavior (cooperative expression) (Phair, 2021). Despite their different theoretical starting points, both approaches converge on a triadic “cognition–affect–behavior” integration and jointly underscore that social-emotional competence spans both intra-individual capacities and interpersonal qualities.

Integrating prior work, the structure of P-SEC can be organized around three core orientations—self-oriented (self-to-self), other-oriented (self-to-others), and task-oriented (self-to-tasks) (Du and Mao, 2018; Schoon, 2021)—each articulated across cognitive, affective, and behavioral levels. The self-oriented domain concerns endogenous development (e.g., gender identity, self-concept), emotion perception and recognition, emotional expression and regulation, and behavioral control. The other-oriented domain reflects internalization of social rules and norms, including social-cue sensitivity, emotion understanding, and prosocial behavior. The task-oriented domain emphasizes symbolic representation of task goals, maintenance of intrinsic motivation, strategies for coping with frustration, and adaptive behavioral regulation (Campbell et al., 2016; Halle and Darling-Churchill, 2016). This framework provides a structured foundation for scale development: the three orientations map onto the core functions of individual development, social relationships, and goal-directed behavior, while the three levels trace progression from implicit cognition and affect to overt behavior—facilitating operationalization into concrete, observable items.

Guided by the Kindergarten Education Guidelines (Trial) and the Guidelines for the Learning and Development of Children Aged 3–6, and drawing on the core factors and exemplar items from the aforementioned instruments validated for Chinese preschoolers, we operationalized young children’s social-emotional competence as comprising several domains: self-recognition and emotion management, interaction and communication with others, and adaptation to the social environment and problem-solving. This framework informed the development of a semi-structured interview protocol.

### 2.2 Qualitative interviews

Using stratified random sampling, we recruited parents of children enrolled in two kindergartens in Nanning across three age bands (3–4, 4–5, and 5–6 years). Twenty-five parents (15 women, 10 men) participated in semi-structured interviews lasting 35–45 min each. After the study aims were explained and informed consent was obtained, all interviews were audio-recorded in full. The interview guide included the following prompts: (1) How well does your child read social cues (i.e., sensitivity to nonverbal and contextual cues)? Which emotions can your child recognize and understand? (2) Which emotions does your child express, and how effectively can they regulate their emotions and behavior? (3) Does your child enjoy interacting with family members, peers, or teachers? How would you describe their interpersonal competence and typical behaviors? (4) Has your child adapted to kindergarten routines? How do they cope with difficulties (e.g., limited self-care skills, conflicts with peers)? (5) Does your child have a positive self-concept? Do they recognize individual differences among children and understand gender differences? Parents were encouraged to provide illustrative examples. Following the predefined protocol, recordings were transcribed verbatim and systematically coded, and a qualitative content analysis was conducted.

### 2.3 Item generation and expert review

Based on prior research and interview coding, we drafted an initial item pool. Six experts (≥10 years’ experience; three kindergarten principals, one psychology professor, one associate professor of early childhood education, and one kindergarten supervisor) rated item-dimension relevance using 5-point Likert scales and provided revisions. Wording was refined, redundancy and ambiguity were reduced, and conceptually distinct items were retained.

### 2.4 Pilot survey

Using purposive sampling, we recruited 20 participants (parents of preschoolers and kindergarten teachers) for a paper-based pretest to evaluate whether the questionnaire items were easily understood and free of ambiguity. This process yielded an initial scale comprising 60 items. Items were rated on a 5-point Likert scale (1 = never observed to 5 = always observed), with higher scores indicating greater social-emotional competence. For items assessing multiple emotions or emotional features (e.g., “Can recognize basic emotions such as happiness, anger, sadness, and fear”), respondents were instructed to assign higher scores if the child could identify the majority (three or more) of the emotions listed.

## 3 Participants and methods

### 3.1 Participants and instruments

The target population comprised typically developing preschoolers aged 36 to 72 months. The scale was completed by primary caregivers—either parents (or other primary guardians) or kindergarten teachers—who had at least 6 months of contact with the children. Respondents rated each child based on observations of consistent, stable behaviors in naturalistic settings rather than on transient events or short-term impressions, ensuring ecological validity. Data were collected via an online platform (Wenjuanxing) during parent-teacher meetings, where caregivers completed the questionnaire on site.

*Sample 1* (item analysis). The 60 items of the initial scale were randomized and uploaded to the Questionnaire Star platform to generate a survey link, which was distributed to parents from two kindergartens in Nanning, Guangxi Zhuang Autonomous Region. A total of 312 questionnaires were returned; after excluding invalid responses (e.g., missing data, patterned responding), 293 valid cases remained (valid response rate = 93.91%). Among the rated children, 150 were boys (51.19%) and 143 were girls (48.81%); the mean age was 4.93 ± 2.08 years.

*Sample 2* (CFA, reliability/validity, and measurement invariance). Using cluster sampling and the same online administration as Sample 1, data were collected from 10 urban and rural kindergartens in Nanning, yielding 1,501 completed questionnaires. After removing invalid responses, 1,373 valid cases were retained (valid response rate = 91.47%). The evaluated children included 739 boys (53.82%) and 634 girls (46.18%), with a mean age of 3.51 ± 1.04 years.

### 3.2 Statistical analyses and data processing

Text data were analyzed in Nvivo 11 using grounded theory with three-stage coding (open, axial, and selective) to construct the scale’s conceptual framework. Using IBM SPSS Statistics 26.0, we conducted item analysis, exploratory factor analysis (EFA), reliability and validity testing, demographic group-difference analyses, and regression analyses on Sample 1. Using IBM SPSS Amos 23.0, we performed confirmatory factor analysis (CFA) and multi-group measurement invariance testing across gender on Sample 2.

## 4 Results

### 4.1 Framework construction

Using Nvivo 11 and the three-stage coding procedures of grounded theory, we conducted a systematic, in-depth analysis of the textual data, adhering to a bottom-up analytic strategy to iteratively extract and integrate key themes. During open coding, 75 preliminary codes were generated from the raw data. In axial coding, based on logical relations and thematic similarity among codes, these were integrated into 11 axial categories: emotion recognition (24 instances), emotion understanding (23), emotion expression (20), self-perception (25), gender role (31), hygiene habits (24), regular routines (28), coping with frustration (22), cooperation and sharing (26), conflict resolution (20), and confronting bullying (10). In selective coding, these were further abstracted into five core categories. Specifically, emotion recognition, understanding, and expression were consolidated as “Emotion Management”; self-perception and gender role as “Self-Concept”; hygiene habits and regular routines as “Daily Habits”; coping with frustration and cooperation/sharing as “Social Interaction”; and conflict resolution together with confronting bullying as “Bullying Counteraction.” Through frequency statistics and progressive abstraction, this categorization reveals the internal logic of the core themes in the data, preliminarily constructs a conceptual framework of preschoolers’ social-emotional competence, and clarifies the relations among categories, thereby providing a theoretical basis for subsequent research.

### 4.2 Item analysis

We first collated 293 valid questionnaires from the initial assessment of preschoolers’ social-emotional competence. Then, based on the total scores across the 60 items, the top 27% and bottom 27% of respondents were selected, and independent-samples *t*-tests were performed on each item. Results indicated significant differences for all items, demonstrating satisfactory item discrimination. Next, item-total correlations were examined; all items showed significant correlations with the total score, ranging from 0.38 to 0.71. Therefore, all 60 items were retained for exploratory factor analysis (EFA).

### 4.3 Exploratory factor analysis

To evaluate factorability, we first conducted Bartlett’s test of sphericity, which was significant, *χ^2^*(1770) = 9,998.262, *p* < 0.001, indicating adequate common variance among items. The Kaiser-Meyer-Olkin (KMO) measure of sampling adequacy was 0.94 (exceeding the 0.70 benchmark), supporting the use of exploratory factor analysis (EFA). We then performed principal components analysis (PCA) with varimax rotation on the 60 items to obtain the rotated factor loading matrix. Item retention followed *a priori* criteria: communality > 0.30; eigenvalue > 1 (Kaiser criterion); primary loading≥0.50 on a single factor (i.e., no salient cross-loadings); at least three items per factor; and the analysis was re-estimated after each item deletion. In total, 35 items were removed. Rotation converged after six iterations, yielding a five-factor solution comprising the remaining 25 items, which formed the scale; the cumulative variance explained was 60.33%. Factor loadings are reported in Table 1.

**Table 1:** Excerpt from exploratory factor analysis of the preschool social-emotional competence scale (*n* = 293).

Item	Rotated loading	Factor
Q1 Recognizes their own basic emotions, such as happiness, anger, sadness, and fear.	0.76	Emotion management
Q2 Recognizes others’ basic emotions (e.g., happiness, anger, sadness, fear) by observing facial expressions, tone of voice, and body language.	0.76	
Q3 Recognizes some complex emotions, such as irritation, jealousy, shame, pride, and frustration.	0.64	
Q4 Understands the reasons for their own basic emotions, such as happiness, anger, sadness, and fear.	0.76	
Q7 Expresses emotions through facial expressions, body movements, or vocal tone.	0.71	
Q14 Clearly knows their own name, age, gender, and distinctive characteristics.	0.78	Self-recognition
Q15 Understands their own preferences and can name things they like.	0.67	
Q26 Selects the appropriate restroom based on their gender, and toileting behaviors are consistent with their gender.	0.63	
Q27 Understands and accepts gender differences in themselves and others; does not exclude peers of the opposite gender during play.	0.71	
Q39 Has regular routines, goes to bed and wakes up early, and does not dawdle in bed.	0.66	Daily habits
Q40 Has good eating habits, eats independently, and is not a picky eater.	0.74	
Q41 Has good personal hygiene habits and is mindful of public hygiene.	0.62	
Q42 Takes care of their own needs independently and has good self-care skills, such as making the bed, organizing their backpack, and tidying toys.	0.62	
Q43 Has good learning habits, such as reading voluntarily and persisting in completing learning tasks.	0.59	
Q45 When encountering setbacks, can express them verbally and attempts to seek help from others.	0.63	Social interaction
Q46 When facing setbacks during group play activities, can politely express difficulties and negotiate with peers (e.g., taking turns, switching roles, letting others try first), reaching consensus to continue play, or transitioning to other activities together if necessary.	0.67	
Q47 Does not give up when facing setbacks; repeatedly tries or adjusts coping strategies until the goal is achieved.	0.71	
Q48 When rejected from joining a game, will imitate peers’ play nearby on their own.	0.68	
Q49 When rejected from joining a game, can find ways to successfully join.	0.72	
Q50 Can find ways to attract peers to play together.	0.68	
Q56 When conflicts arise with peers, typically negotiates and uses reasonable methods (e.g., rock-paper-scissors) to resolve conflicts.	0.70	
Q57 When others have different opinions, can listen to and accept others’ views; when disagreeing, provides reasons.	0.71	
Q58 When hit by another child, will immediately retaliate physically.	0.79	Bullying counteraction
Q59 When bullied, will loudly warn the aggressor.	0.81	
Q60 When bullied, will seek help from others.	0.56	

### 4.4 Validity analysis

#### 4.4.1 Construct validity

In Sample 2 (*n* = 1,373), a confirmatory factor analysis (CFA) was performed on the five-factor model. Although the revised model showed excellent overall fit, issues with discriminant validity arose. Specifically, the correlation between Emotion Management and Self-recognition exceeded 0.85, suggesting a high degree of overlap between these two dimensions. Because each dimension must retain at least three items, a higher-order factor was introduced to better explain the shared variance between Emotion Management and Self-recognition (Zhang et al., 2022).

Model testing strategy. Sample 2 (*n* = 1,373) was randomly partitioned into Sample 2a (*n* = 773) and Sample 2b (n = 600). CFA was conducted in AMOS on Sample 2a, and Sample 2b served as an independent dataset for a follow-up EFA to probe the structure of the best-fitting model. Guided by the magnitude of inter-factor correlations, five competing specifications were evaluated:

Model 1 (one-factor model): All items loading on a single latent factor.
Model 2 (two-factor model): (Emotion Management + Self-recognition + Daily Habits), (Social Interaction + Bullying Counteraction).
Model 3 (three-factor model): (Emotion Management + Self-recognition), (Daily Habits + Social Interaction), and Bullying Counteraction.
Model 4 (four-factor model): (Emotion Management + Self-recognition), Daily Habits, Social Interaction, and Bullying Counteraction.
Model 5 (five-factor model): Emotion Management, Self-recognition, Daily Habits, Social Interaction, and Bullying Counteraction as distinct factors.

Fit indices for the revised versions of each model are reported in Table 2.

**Table 2:** Comparison of model fit indices across competing models (*n* = 773).

Model	χ*^2^/df*	GFI	CFI	AGFI	IFI	TLI	RMSEA
Single-factor	9.93^***^	0.79	0.85	0.72	0.85	0.82	0.12
Two-factor	5.71^***^	0.90	0.92	0.86	0.93	0.91	0.09
Three-factor	5.39^***^	0.90	0.92	0.86	0.92	0.91	0.08
Four-factor	3.86^***^	0.92	0.95	0.89	0.95	0.94	0.06
Five-factor	4.91^***^	0.95	0.97	0.93	0.97	0.96	0.05

^*^p < 0.05, ^**^p < 0.01, ^***^p < 0.001.

**Figure 1:**
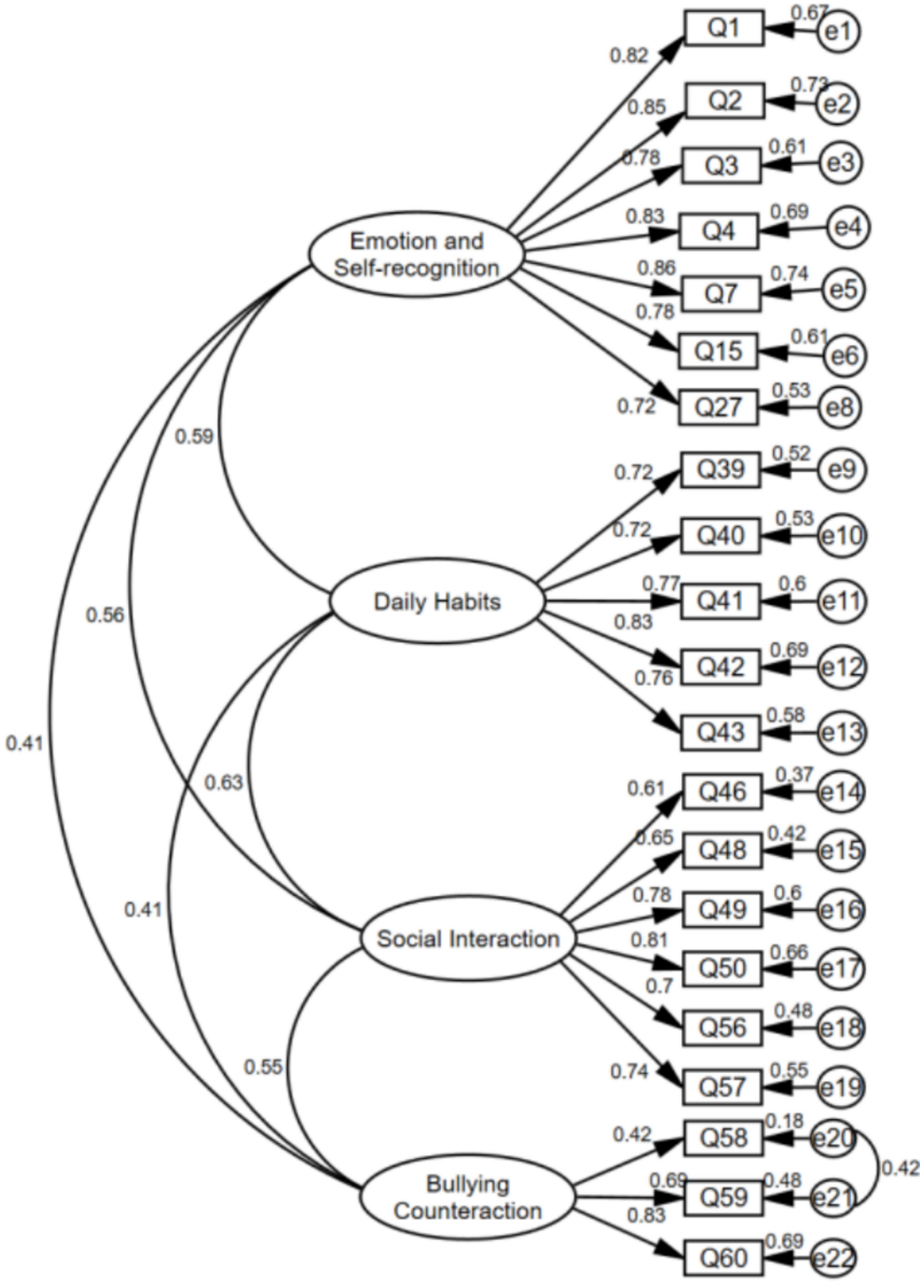
Confirmatory factor analysis (CFA) path diagram for the revised four-factor model.

After model comparisons, only the four-factor model—once revised—met the target criteria. Specifically, indicators with cross-loadings (Q45, Q47) were removed, and items exhibiting large error-term modification indices (MI > 50; Q14, Q26) were also deleted. The resulting solution showed CFI and IFI values closest to 0.95, indicating a superior overall fit and providing a solid basis for subsequent tests of measurement invariance (Byrne, 2016). The CFA path diagram is presented in Figure 1. For the 21 retained items, standardized factor loadings on their respective dimensions ranged from 0.42 to 0.86, indicating strong indicator contributions to their latent factors.

Furthermore, within the Bullying Counteraction dimension, although the residuals of Q58 and Q59 are correlated and the standardized regression weight of Q58 is substantially lower than those of Q59 and Q60, the possibility that parents may educate their children to retaliate directly against bullies after being victimized cannot be ruled out. This is particularly plausible given the increasing emphasis contemporary parents place on self-protection awareness (Marlow et al., 2023). Consequently, Q58 has been retained.

A second exploratory factor analysis (EFA) was conducted on the independent split sample Sample 2b (*n* = 600) to further probe the structure supported by the CFA. With principal components extraction and Varimax rotation, diagnostics indicated excellent factorability: KMO = 0.96; Bartlett’s test χ^2^ = 9392.07, df = 210, *p* < 0.001. All 21 items showed factor loadings > 0.50, with communalities ranging 0.56–0.83; the solution accounted for 71.67% of the total variance (see Table 3), an increase of 11.34 percentage points over the first EFA. In this structure, Emotion Understanding and Self-recognition merged into a single factor—Emotion and Self-recognition—characterized by children’s recognition, understanding, and expression of their own and others’ emotions.

**Table 3:** Excerpt from the exploratory factor analysis of the preschool social-emotional competence scale (*n* = 600).

Item	Factor				Communality
	Emotion and self-recognition	Daily habits	Social interaction	Bullying counteraction	
Q1	0.85				0.81
Q2	0.84				0.82
Q3	0.72				0.68
Q4	0.80				0.78
Q7	0.87				0.83
Q15	0.82				0.76
Q27	0.72				0.68
Q39		0.71			0.65
Q40		0.77			0.74
Q41		0.65			0.71
Q42		0.75			0.74
Q43		0.67			0.68
Q46			0.57		0.56
Q48			0.76		0.64
Q49			0.75		0.72
Q50			0.7		0.70
Q56			0.69		0.67
Q57			0.64		0.69
Q58				0.84	0.76
Q59				0.76	0.77
Q60				0.53	0.67
Cumulative variance explained	51.35%	60.95%	66.91%	71.67%	

#### 4.4.2 Convergent and discriminant validity

Composite reliability (CR) values for all dimensions were≥0.70, indicating satisfactory internal consistency among indicators within each factor. The average variance extracted (AVE), which reflected the proportion of variance in the indicators accounted for by their latent factor, ranged from 0.45 to 0.65. Although the AVE value for Bullying Counteraction was slightly below the conventional cutoff of 0.50, all CR values exceeded 0.70, suggesting acceptable convergent validity overall. For discriminant validity, the square root of AVE for each factor exceeded its largest inter-factor correlation, consistent with the Fornell–Larcker criterion. For example, the square root of AVE for Emotion and Self-recognition was 0.81, greater than its correlations with other factors (|r| = 0.40–0.59). In addition, the heterotrait–monotrait ratio (HTMT) values ranged from 0.51 to 0.73, all below the conservative threshold of 0.85, providing further evidence of discriminant validity (Henseler et al., 2015)(see Table 4). Taken together, these results supported acceptable to good discriminant validity for Daily Habits, Social Interaction, and Bullying Counteraction as well (see Table 4, 5). Although some inter-factor correlations were moderate, this pattern was theoretically expected because the four dimensions represent related facets of preschool social-emotional competence rather than entirely unrelated traits. This interpretation is also consistent with the conceptual distinctions discussed later for Emotion and Self-recognition, Daily Habits, Social Interaction, and Bullying Counteraction.

**Table 4:** Heterotrait-monotrait ratio (HTMT) of the four constructs (*n* = 773).

Construct dimension	Emotion and self-recognition	Daily habits	Social interaction	Bullying counteraction
Emotion and self-recognition	–			
Daily habits	0.66	–		
Social interaction	0.63	0.73	–	
Bullying counteraction	0.51	0.52	0.69	–

All HTMT values were below 0.85, supporting discriminant validity.

**Table 5:** Convergent validity, discriminant validity, and inter-factor correlations of the four-dimension preschool social-emotional scale (*n* = 773).

Construct dimension	CR	AVE	Total score	Emotion and self-recognition	Daily habits	Social interaction	Bullying counteraction
Emotion and self-recognition	0.93	0.65	0.85^***^	0.81			
Daily habits	0.87	0.58	0.82^***^	0.59^***^	0.76		
Social interaction	0.86	0.52	0.85^***^	0.56^***^	0.63^***^	0.72	
Bullying counteraction	0.70	0.45	0.65^***^	0.41^***^	0.41^***^	0.55^***^	0.67

Diagonal elements are the square roots of AVE. ^***^p < 0.001.

#### 4.4.3 Reliability testing

The revised total scale showed a Cronbach’s *α* of 0.94 and a split-half reliability of 0.83, indicating high internal consistency for the instrument as a whole. Cronbach’s α coefficients for the four dimensions—Emotion and Self-recognition, Daily Habits, Social Interaction, and Bullying Counteraction—ranged from 0.60 to 0.94. In addition, given the multidimensional structure of the scale, McDonald’s omega coefficients were calculated for the total scale and each subscale, as omega has been recommended as a complementary estimate of internal consistency to Cronbach’s alpha (Malkewitz et al., 2023). The omega coefficient was 0.94 for the total scale and ranged from 0.86 to 0.94 across the four dimensions, further supporting the internal consistency of the revised four-factor model (see Table 6).

**Table 6:** Reliability testing of the preschool social-emotional competence scale (*n* = 773).

Reliability index	Total scale	Emotion and self-recognition	Daily habits	Social interaction	Bullying counteraction
Cronbach’s α	0.94	0.94	0.88	0.87	0.76
Split-half reliability	0.86	0.88	0.83	0.86	0.60
McDonald’s ω	0.94	0.94	0.91	0.90	0.86

#### 4.4.4 Measurement invariance across gender

Using AMOS and a stepwise nested-model approach, the four-factor measurement model of social-emotional competence was tested for cross-group (male vs. female) measurement invariance with the entire Sample 2 (Zhao, 2007). Across all models, fit indices were favorable (GFI ≥ 0.90, CFI ≥ 0.94, RMSEA≤0.05), indicating good overall model fit. Because the models were nested, measurement invariance was evaluated primarily on the basis of changes in approximate fit indices rather than the chi-square difference test alone, given the sensitivity of χ^2^ to sample size. Following widely used recommendations for measurement invariance testing, ΔCFI ≤ 0.01 was taken as the primary criterion for invariance, with ΔRMSEA ≤ 0.015 used as an additional reference standard (Chen, 2007; Cheung and Rensvold, 2002). First, Model 1 (the unconstrained baseline model) was compared with Model 2 (metric invariance: equality constraints on factor loadings). Results showed Δχ^2^ = 19.05 with df = 17,and ΔCFI = 0.00 and ΔRMSEA = 0.00, indicating that Model 2 fit as well as Model 1; thus, the more parsimonious Model 2 was accepted, supporting equality of factor loadings across gender. Subsequently, Model 2 was compared with Model 3 (equality of “structural weights,” with equality constraints imposed on the regression coefficients of first-order factors), Model 3 with Model 4 (equality of “structural covariances,” with equality constraints imposed on the covariance of the total score of socio-emotional competence), Model 4 with Model 5 (equality of “structural residuals,” with equality constraints imposed on the residuals of first-order factors), and Model 5 with Model 6 (equality of “measurement residuals,” with equality constraints imposed on the measurement error variances), as detailed in Table 7. Across these successive comparisons, CFI remained stable at 0.94 and RMSEA showed only negligible change (from 0.05 to 0.04), supporting invariance across increasingly constrained models. Accordingly, the most parsimonious Model 6 was retained. These findings indicate that the social-emotional competence measurement model demonstrated configural invariance, metric invariance, and progressively stricter forms of invariance across gender among preschoolers.

**Table 7:** Fit indices for the baseline and invariance models (*n* = 1,373).

Model	χ*^2^*	*df*	GFI	CFI	RMSEA	△χ*^2^*	△*df*	△GFI	△CFI
Model 1	1430.51^***^	368	0.91	0.94	0.05				
Model 2	1449.56^***^	385	0.90	0.94	0.05	19.05	17	0.01	0.00
Model 3	1453.60^***^	388	0.90	0.94	0.05	4.05	3	0.00	0.00
Model 4	1453.87^***^	389	0.90	0.94	0.05	0.26	1	0.00	0.00
Model 5	1464.37^***^	393	0.90	0.94	0.05	10.50	4	0.00	0.00
Model 6	1500.17^***^	414	0.90	0.94	0.04	35.80	22	0.00	0.00

### 4.5 Differences in demographic variables

#### 4.5.1 Differences in gender and only-child status

To investigate the differences in social–emotional competence and its dimensions based on children’s gender and only-child status, this study utilized an independent samples t-test for analysis (see Table 7). The results indicate that boys and girls show significant differences in Emotion and Self-recognition and Daily Habits (*p* < 0.05), with girls scoring higher. However, no significant differences were found in social interaction, Bullying Counteraction, or the overall social-emotional competence score (*p* > 0.05). Furthermore, no significant differences were observed between only children and non-only children in terms of overall social-emotional competence or its dimensions (*p* > 0.05).

#### 4.5.2 Differences in family socio-economic status

Family socio-economic status (SES) is divided into objective family socio-economic status (Objective-SES) and subjective family socio-economic status (Subjective-SES). Objective-SES refers to material resources and social capital, quantifiable through dimensions such as education level and income (Zhou et al., 2018). Subjective-SES is defined as an individual’s subjective understanding and perception of their relative social position (Kraus et al., 2012), assessed through the Arthur subjective social status “ladder self-assessment” method (Adler et al., 2000). Based on the scores, participants were divided into high and low SES groups by ranking the top and bottom 27% of the sample. One-way analysis of variance (ANOVA) was used to explore the differences in social-emotional competence across various SES levels (see Table 8).

**Table 8:** Differences in social-emotional competence based on gender and only-child status (*n* = 1,373).

Dimension	Boys (*n* = 739)	Girls (*n* = 634)	t	Only-child (*n* = 371)	Non-only-child (*n* = 1,002)	t
Emotion and self-recognition	29.89 ± 5.22	30.56 ± 4.90	−2.4^*^	30.32 ± 4.85	30.15 ± 5.17	0.55
Daily habits	18.38 ± 3.80	18.89 ± 3.78	−2.47^*^	18.33 ± 3.56	18.72 ± 3.88	−1.67
Social interaction	21.01 ± 4.33	21.16 ± 4.50	−0.65	20.72 ± 4.08	21.21 ± 4.52	−1.92
Bullying counteraction	10.70 ± 2.34	10.47 ± 2.52	1.72	10.42 ± 2.40	10.65 ± 2.43	−1.56
Social-emotional competence	79.98 ± 13.09	81.07 ± 12.98	−1.55	79.80 ± 12.00	80.74 ± 13.41	−1.18

In terms of subjective-SES, the results showed significant differences across the three levels of subjective-SES in Emotion and Self-recognition, Daily Habits, social interaction, Bullying Counteraction, and overall social-emotional competence (*p* < 0.01). *Post hoc* LSD tests revealed that the low subjective-SES group scored significantly lower than the medium and high subjective-SES groups in terms of social-emotional competence and its dimensions (*p* < 0.01). However, no significant difference was found between the medium and high subjective-SES groups.

In terms of objective-SES, significant differences were observed between groups for Emotion and Self-recognition and Bullying Counteraction (*p* < 0.01), while no significant differences were found in Daily Habits and social interaction (*p* > 0.05). Further Post hoc LSD tests showed that the low objective-SES group scored significantly lower than both the medium and high objective-SES groups in Emotion and Self-recognition (MD = −1.76, *p* < 0.01; MD = −2.08, *p* < 0.01). In Bullying Counteraction, significant differences were found between the low and medium objective-SES groups (MD = −0.42, *p* < 0.01) as well as between the low and high objective-SES groups (MD = −0.53, *p* < 0.01), but no significant difference was found between the medium and high objective-SES groups (see Table 9).

**Table 9:** Differences in social-emotional competence based on family socio-economic status.

Dimension	Objective family socio-economic status			*F*	Subjective family socio-economic status			*F*
	1.0 (*n* = 378)	2.0 (*n* = 592)	3.0 (*n* = 403)		1.0 (*n* = 389)	2.0 (*n* = 550)	3.0 (*n* = 434)	
Emotion and self-recognition	28.83 ± 5.57	30.59 ± 4.47	30.91 ± 5.23	19.87^***^	29.09 ± 5.53	30.55 ± 4.87	30.75 ± 4.79	13.34^***^
Daily habits	18.38 ± 3.96	18.66 ± 3.64	18.76 ± 3.86	1.08	17.76 ± 3.75	18.96 ± 3.73	18.94 ± 3.80	14.2^***^
Social interaction	20.75 ± 4.57	21.24 ± 4.36	21.15 ± 4.31	1.48	20.10 ± 4.36	21.47 ± 4.43	21.47 ± 4.29	13.71^***^
Bullying counteraction	10.26 ± 2.60	10.67 ± 2.36	10.78 ± 2.32	5.24^**^	10.28 ± 2.42	10.63 ± 2.46	10.82 ± 2.36	5.12^**^
Social-emotional competence	78.22 ± 14.07	81.17 ± 12.20	81.60 ± 13.01	8.06^***^	77.23 ± 13.19	81.61 ± 12.85	81.98 ± 12.68	17.44^***^

1.0 indicates low level, 2.0 indicates medium level, and 3.0 indicates high level.

## 5 Discussion

The scale was developed with reference to the local culture of Guangxi Zhuang Autonomous Region, China, and examines the content structure of P-SEC. Grounded in three core relational structures—self-to-self, self-to-other, and self-to-task/event—crossed with three functional levels (cognition-affect-behavior), we constructed a relatively comprehensive theoretical framework. Exploratory factor analysis (EFA) and confirmatory factor analysis (CFA) yielded four factors: Emotion and Self-recognition, Social Interaction, Daily Habits, and Bullying Counteraction.

### 5.1 Factor 1

Emotion and Self-recognition integrates theories of emotion recognition and self-development. Drawing on Denham’s framework of emotion understanding (Denham, 1986), it emphasizes children’s ability to identify and understand emotions in themselves and others—a foundation for emotional socialization. This factor encompasses both emotion recognition and self-knowledge. Unlike the OECD framework’s focus on emotion control, it places greater weight on the identification and understanding of one’s own and others’ emotional states. Research indicates that children aged 3–6 cannot yet recognize complex emotions rapidly and accurately (Alwaely et al., 2021), though they can regulate their emotions with appropriate guidance. Accordingly, assessment of emotional development in social contexts should be calibrated to developmental characteristics. The self-knowledge component draws on Lewis’s theory of self-awareness development, focusing on emerging gender-role cognition—an important marker of self-awareness development and socialization (Lewis et al., 1989; Liu, 2024)—indexed by simple daily behaviors (e.g., choosing a restroom aligned with one’s gender; not excluding peers of another gender during play) that signal the internalization of gender roles. This conceptualization differs from the CASEL Wheel’s “self-awareness,” which emphasizes higher-order capacities such as understanding one’s thoughts and values, cultural background, and language abilities (CASEL, 2020).

### 5.2 Factor 2

Daily Habits is grounded in self-regulation theory. Kopp noted that early childhood is a critical period for the development of self-control, and adherence to rules regarding diet, daily routines, and hygiene represents observable manifestations of self-control (Kopp, 1982). Accordingly, this factor primarily concerns diet, daily routines, and hygiene. The possession of good daily habits reflects not only self-control but also the internalization of social rules; it implies that children can suppress immediate impulses and forego lower-value goals, comply with rules autonomously, and thereby foster social adaptation (Shen, 2021). In China’s collectivist context, the normative character of Daily Habits is particularly salient. By contrast, Western contexts place greater emphasis on individual responsibility and the right to autonomous choice, illustrating how culture shapes behavioral patterns (Phair, 2021). Accordingly, when constructing the scale, items assessing Daily Habits were culturally adapted for Chinese preschoolers while maintaining an accurate representation of observable behavioral norms.

### 5.3 Factor 3

Social Interaction. Items are largely situated in play contexts and occur primarily during peer play. Such simulated social situations promote emotional intelligence, the formation of emotional bonds (e.g., friendship), and the development of positive social qualities (Chen, 2023; Li and Zhao, 2009; Niu and Ran, 2023). Consistent with embodied cognition theory, cognitive development is rooted in the interaction between body and environment, and social interaction during play serves as a vehicle for young children’s social-cognitive development. Play not only advances social development but also provides a context in which the social skills acquired—whether they help children respond constructively to adverse situations and actively cooperate with others in safe environments—serve as observable indicators of the level and trajectory of social-emotional competence (Jia et al., 2025).

### 5.4 Factor 4

Bullying Counteraction, draws on the Olweus Bullying Prevention Program, which emphasizes that teaching young children to identify and respond to bullying is an effective strategy for protecting their mental health (Olweus and Limber, 2010). Accordingly, this factor focuses on whether children can employ effective strategies when confronted with bullying—for example, verbal intervention, physical self-defense, or seeking external assistance—to stop the bullying behavior. The prominence of resources such as “early childhood bullying prevention websites” indicates growing attention to peer bullying in early childhood; as a risk factor for mental health, bullying requires children to acquire counter-bullying strategies through social learning, lest long-term mental health and social adaptation be compromised (Armitage, 2021; Ni et al., 2024). This supplementary dimension highlights culturally and institutionally congruent pathways, notably polite assertiveness and help-seeking from authority figures.

Reliability testing showed that the total scale demonstrated excellent internal consistency (Cronbach’s *α* = 0.94) and split-half reliability (0.83), with all subscales exhibiting reliability coefficients above 0.60, indicating good reliability (Loewenthal and Lewis, 2020). Item analysis, exploratory factor analysis (EFA), and confirmatory factor analysis (CFA) further indicated that, after removing items with low loadings or cross-loadings, the remaining 21 items formed a four-factor model. The revised model achieved good fit, with GFI, CFI, and TLI all exceeding 0.90, and demonstrated solid convergent and discriminant validity, suggesting that the distinct dimensions capture different facets of preschoolers’ social-emotional competence. Finally, multi-group measurement invariance across gender—covering measurement weights, structural weights, structural covariances, structural residuals, and measurement errors—was supported, indicating equivalent measurement structure and reference units for boys and girls and permitting mean-level comparisons across dimensions (Muthén and Asparouhov, 2018). In sum, the scale meets core psychometric standards.

Results from the differential test indicate that girls significantly outperform boys in emotion and self-recognition and daily habits, which aligns with existing research that suggests girls have advantages in emotional sensitivity and self-regulation (Hosokawa and Katsura, 2017). It is worth noting that no significant differences were found between only children and non-only children in the development of social-emotional competence. This is consistent with recent research on Chinese families, which indicates that the only-child group does not show disadvantages in social-emotional development due to family structure characteristics. On the contrary, they may benefit from enhanced development due to greater parental resource investment (Qianqian et al., 2020).

In exploring the relationship between family socio-economic status (SES) and factors associated with young children’s social-emotional competence, the analysis revealed that children with low subjective SES scored significantly lower in social-emotional competence compared to those with medium and high subjective SES. However, no significant differences were observed between the medium and high subjective SES groups. Although young children have not yet developed an abstract understanding of social class, they perceive their social standing through family environment and parenting styles. Children with low subjective SES, sensing social disadvantage, often face greater psychological stress, which negatively impacts their emotional regulation and social skills development (Wang and Huang, 2024). These children tend to exhibit lower emotional awareness and self-regulation due to feelings of relative deprivation. Additionally, they may be at a disadvantage in Daily Habits and social interactions because of insufficient resources and psychological capital, and they lack effective coping strategies when dealing with conflict or bullying (Greitemeyer and Sagioglou, 2018). In contrast, objective SES is associated with dimensions of social-emotional competence, such as Emotion and Self-recognition, Bullying Counteraction, and others. This suggests that the lack of material resources and increasing environmental stress may directly weaken children’s emotional regulation and resilience (Wu et al., 2020). Gautam emphasized the role of parenting styles and parent–child relationships in explaining inequalities in children’s mental health, noting their close relationship with SES (Gautam et al., 2024). High-objective SES families typically provide richer educational resources and more stable home environments, such as parents’ digital literacy and access to digital resources, which influence children’s use of technology and, consequently, their learning, social, and cognitive development. On the other hand, children from low-objective SES families are more likely to face risks such as malnutrition and developmental delays, which can impair cognitive and emotional development (Chang et al., 2024). However, no differences were observed between objective SES groups in terms of Daily Habits and social interaction, which contrasts with the findings for subjective SES. This may suggest that social-emotional behaviors, such as Daily Habits and social interactions, are more influenced by family practices, cultural customs, and individual personality traits rather than being directly determined by economic conditions (Highlander and Jones, 2022; Mistry et al., 2021).

## 6 Implications and future directions

This scale provides a measurement tool for in-depth research on young children’s social-emotional competence in China. It not only helps gauge the developmental level of children’s core competencies but also enables the investigation of potential determinants of social-emotional competence, thereby offering scientific evidence to inform current educational practices and providing a theoretical foundation and empirical support for preschool education reform. Application of this scale will facilitate a comprehensive understanding of the developmental processes underlying P-SEC cognition and behavioral formation, thereby suggesting avenues for home-school collaboration: for example, leveraging peer play and teacher-child interactions to enhance empathy and cultivate sound daily habits; and using structured scenarios and role-play to strengthen children’s psychological resilience in the face of developmental setbacks, everyday difficulties, and bullying situations—thus promoting holistic mental health development.

The present tool assesses P-SEC from a single-informant, other-report perspective and does not incorporate context-sensitive, longitudinal indicators within specific situations, which constitutes a limitation. This scale is designed to help educators and parents understand the basic status of children’s social-emotional development and to provide reference for family upbringing and preschool education. It is important to emphasize, however, that this scale is not intended for medical diagnosis or clinical screening. Future research could adopt multi-perspective approaches by integrating context-based assessments to evaluate children’s social-emotional competence, while simultaneously refining item construction to reduce double-barreled content. In addition, further efforts should be made to improve longitudinal measurement methods to construct the dynamic developmental trajectories of children’s social-emotional competence across different early childhood periods, thereby deepening the understanding of its underlying developmental regularities.

## Data Availability

The raw data supporting the conclusions of this article will be made available by the authors, without undue reservation.
